# Tea-induced calmness: Sugar-sweetened tea calms consumers exposed to acute stressor

**DOI:** 10.1038/srep36537

**Published:** 2016-11-16

**Authors:** Shilpa. S. Samant, Katherine Wilkes, Zephania Odek, Han-Seok Seo

**Affiliations:** 1 Department of Food Science, University of Arkansas, 2650 North Young Avenue, Fayetteville, AR 72704, USA

## Abstract

The food and beverage industry has been increasingly replacing sugar with non-nutritive sweeteners in their sweetened products to control or reduce total calories. Research comparing the effect of nutritive and non-nutritive sweeteners on emotional state of participants exposed to acute stressors is still limited. This study aimed to determine the effect of drinking tea sweetened with either a nutritive sweetener (sugar) or a non-nutritive sweetener (sucralose or stevia) on emotional state, in terms of calmness and pleasantness, of participants exposed to an acute stressor. Effects of acute stress on sweetness intensity and overall liking of tea beverages were also determined. Results showed that the possibility of tea-induced calmness, calculated as the difference between calmness ratings after and before drinking a tea sample, was established on stress session in the sugar-sweetened tea. Overall liking, but not the sweetness intensity, of the sugar-sweetened tea was affected by acute stress. In conclusion, this study provides empirical evidence that the consumption of tea sweetened with nutritive sweetener, but not with non-nutritive sweetener, has calming effect on consumers with acute stress, suggesting that this effect may not be due to the sweet taste of sugar, but due to the caloric nature of the sweetener.

Acute stress, according to the American Psychological Association, is the most common form of stress. Characteristically, acute stress is short-term and is caused mostly by a situation/condition of demands and pressures in either the recent past or the immediate future that impacts an individual’s current emotional state. Symptoms of acute stress include aspects of “emotional distress” such as anxiety, irritation, and/or anger^1^. Acute stress can negatively impact the human body in many ways, including its taste functions. Research shows that acute stress, whether it is mental or physical in nature, has the potential to affect taste perception in humans^2^,^3^,^4^. One such experiment in Japan examined bitter, sour, and sweet taste intensity perception under conditions of both acute mental (letter search) and physical (exercise) stress^2^. Interestingly, reduction in the total amount of taste (i.e., area under the curve of the time-intensity analysis) was observed for all three taste qualities in the presence of mental stress, although no reduction in the maximum taste intensity was observed for sweet and sour tastes under mental stress. Such reduction in the total amount of taste was not obtained for bitter and sweet taste qualities when the participants were exposed to physical stress, indicating that the effect of acute stress on sweet taste perception may vary by the type of stressor (i.e., mental versus physical stress)^2^. In another study, participants were exposed to acute mental stress condition such as public speaking or solving a math problem^3^. In their study, participants’ sweet taste intensity decreased under stress conditions relative to perceived sweet taste intensity under a non-stress condition^3^. These observations suggest that in addition to the physical structure of food, the physiological and psychological conditions of the person tasting the food have a substantial impact on an individual’s taste perceptions, especially with respect to sweet taste.

Most of the above-mentioned research regarding acute stress and sweet taste perception has been restricted to the use of sucrose (sugar) as the taste-imparting agent. However, popular non-nutritive sweeteners currently used as sugar substitutes must also be considered. The use of non-nutritive sweeteners dates back to 1879 when Remsen and Fahlberg developed saccharin, the first non-nutritive sweetener^5^. Since then the use of non-nutritive has been greatly on the rise. Weihrauch and Diehl^6^ assert that every citizen in Western countries has most likely consumed non-nutritive sweeteners at one point or another, either knowingly or unknowingly. As of today, the Food and Drug Administration (FDA) has approved five chemical substances as non-nutritive sweeteners; these include sucralose, neotame, acesulfame-K, aspartame, and saccharin. Additionally, recently FDA has recognized stevia as a sugar-substitute and dietary supplement with “GRAS” (generally recognized as safe) status^7^. These non-nutritive sweeteners, when compared to sucrose, have negligible or no caloric value as well as producing more intense sweetness sensation. For example, sucralose is 600 times sweeter than sucrose^8^. These properties make non-nutritive sweeteners ideal for inclusion in diet or low sugar products^9^. No concrete evidence to date calls non-nutritive sweeteners into question^10^, but public perception views “unnatural” artificial sweeteners as potentially detrimental to human health. In the literature, these non-nutritive sweeteners have been referred to as high intensity sweeteners, non-sucrose sweeteners, intense sweeteners, and sugar-free sweeteners^11^. In this paper, the term “non-nutritive sweeteners” will be used exclusively and sucrose henceforth will be referred to as “sugar”.

There are a few studies wherein emotional responses induced by non-nutritive sweeteners have been compared with those induced by sugar, using implicit (e.g., emotional term questionnaire) and explicit (e.g., facial expression response) methods^12^. However, to the best of the authors’ knowledge, no previous study has directly compared the effects of nutritive and non-nutritive sweeteners on emotional state, such as calmness and pleasantness, when exposed to an acute stressor. This study aimed at comparing the effects of both nutritive (sugar) and non-nutritive (stevia and sucralose) sweeteners on the emotional state of participants exposed to acute stressor, in terms of tea induced calmness and pleasantness. Previous studies have shown that a person’s emotional state is comprised of two attributes: “arousal” (e.g., calming versus exciting) and “valence” (e.g., pleasant versus unpleasant)^13^. Thus, this study was designed to elucidate the effect of sweeteners on both arousal and valence factors of emotional state. In addition, the impacts of acute stress on sweet taste perception of both sugar and non-nutritive sweeteners have not been studied. Therefore, this study aimed at determining the effects of acute stress on sweetness intensity and overall liking of both nutritive and non-nutritive sweeteners to explore whether these aspects can mediate the possible effects of sweeteners on emotional state under acute stress.

## Results

### Profile of Mood States (POMS) ratings

#### Mean anxiety rating comparison between no-stress day and stress day

On stress day, participants were asked to take an intelligence quotient (IQ) test within a time limit of 10 minutes to induce acute mental stress. Participants were asked to complete an abridged version of a Profile of Mood States (POMS) questionnaire^14^ to measure their mood state on no-stress and stress days before and after the IQ test.

Participants were found to be significantly more anxious (in terms of mean anxiety ratings) on stress day after the IQ test [mean ± standard deviation (SD) = 1.74 ± 0.57] than on no-stress day (1.48 ± 0.31) (*P* = 0.001). Additionally, participants’ baseline rating on stress day (before the stressor was provided; mean ± SD = ±1.48 ± 0.23) was not different from their ratings on no-stress day (*P* = 0.80), but was significantly lower than the rating after the stressor was provided on stress day (*P* = 0.001). This implies that the stressor used in this study (i.e., a timed IQ test) was successful in inducing acute stress in the participants.

#### POMS rating comparison between no-stress day and stress day for positive and negative moods

**Figure 1:**
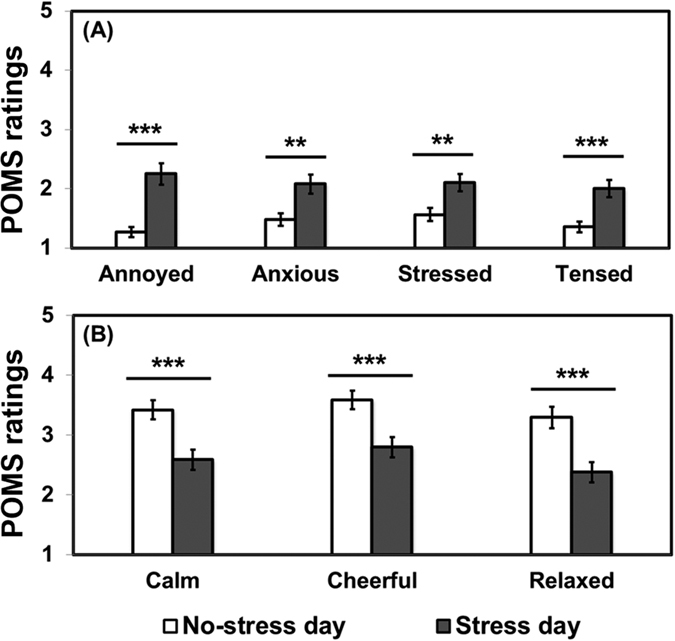
(**A**) Comparisons of POMS rating of individual negative moods (annoyed, anxious, stressed, tensed) on “No-stress day” and “Stress day”. (**B**) Comparison of POMS rating of individual positive moods (calm, cheerful, relaxed) between “No-stress day” and “Stress day”. A Wilcoxon Signed-Rank test was used for data analysis. **and ***represent a significant difference at *P* < 0.01 and *P* < 0.001, respectively. Error bars represent standard error of mean.

To further verify the efficiency of our method of inducing acute stress into participants, we compared POMS ratings on certain positive and negative moods. Interestingly, as shown in Fig. 1(A), it was found that participants felt more annoyed (*P* < 0.001), anxious (*P* = 0.003), stressed (*P* = 0.001), and tensed (*P* < 0.001) on stress day after the IQ test than on no-stress day. In contrast, participants’ ratings were significantly higher on no-stress day than on stress day after the IQ test for positive moods such as calm (*P* < 0.001), cheerful (*P* < 0.001), and relaxed (*P* < 0.001) as shown in Fig. 1(B).

### Tea-induced calmness comparison between no-stress day and stress day

**Figure 2:**
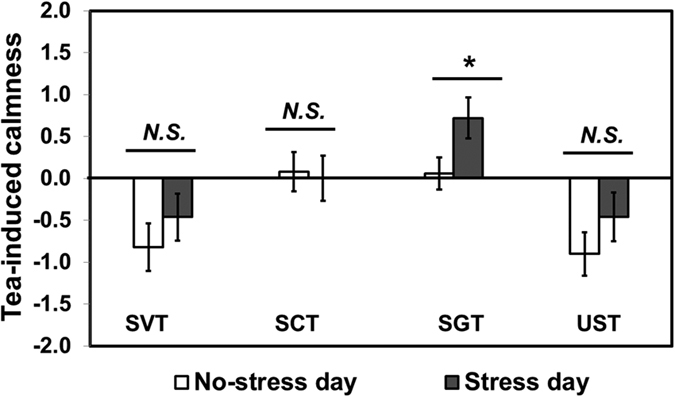
Comparisons of tea-induced calmness between “No-stress day” and “Stress day” for stevia-sweetened tea (SVT), sucralose-sweetened tea (SCT), sugar-sweetened tea (SGT), and unsweetened tea (UST). Tea-induced calmness is calculated as the difference between calmness ratings after and before drinking tea sample. A Wilcoxon Signed-Rank test was used for data analysis. *N.S.* represents no significant difference at *P* < 0.05. *Represents a significant difference at *P* < 0.05. Error bars represent standard error of mean.

Tea-induced calmness was calculated as the difference between calmness ratings after and before drinking each of the following tea samples: stevia-sweetened tea (SVT), sucralose-sweetened tea (SCT), sugar-sweetened tea (SGT), and unsweetened tea (UST). SGT-induced calmness was found to be higher on stress day compared to on no-stress day (*P* = 0.02) (Fig. 2). However, no significant differences were found for any other tea sample (*P* > 0.05, for all).

### Tea-induced pleasantness comparison between tea samples

**Figure 3:**
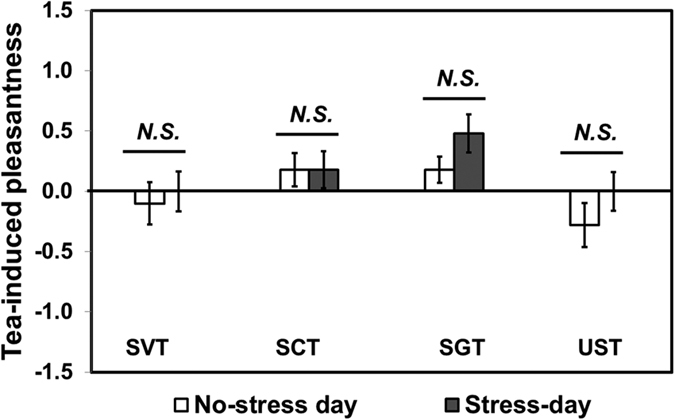
Comparisons of tea-induced pleasantness between “No-stress day” and “Stress day” for stevia-sweetened tea (SVT), sucralose-sweetened tea (SCT), sugar-sweetened tea (SGT), and unsweetened tea (UST). Tea-induced pleasantness is calculated as the difference between calmness ratings after and before drinking tea sample. A Wilcoxon Signed-Rank test was used for data analysis. *N.S.* represents no significant difference at *P* < 0.05. Error bars represent standard error of mean.

Tea-induced pleasantness was calculated as the difference between pleasantness ratings after and before drinking a tea sample. As shown in Fig. 3, sweetened tea-induced pleasantness was not significantly different between no-stress day and stress day for any of the tea samples (*P* > 0.05, for all).

### Comparison of sweetness intensity of tea samples between no-stress day and stress day

**Figure 4:**
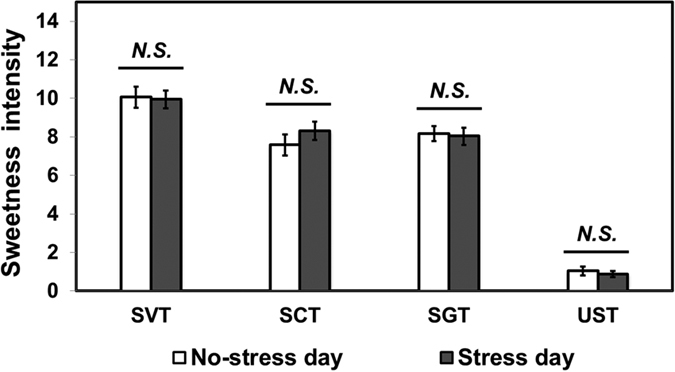
Comparisons of sweetness intensity between “No-stress day” and “Stress day” for stevia-sweetened tea (SVT), sucralose-sweetened tea (SCT), sugar-sweetened tea (SGT), and unsweetened tea (UST). A Wilcoxon Signed-Rank test was used for data analysis. *N.S.* represents no significant difference at *P* < 0.05. Error bars represent standard error of mean.

Figure 4 shows that no significant differences were found in sweetness intensity for any of the four tea samples when no-stress day and stress day ratings were compared (*P* > 0.05, for all).

### Comparison of overall liking of tea samples between no-stress day and stress day

**Figure 5:**
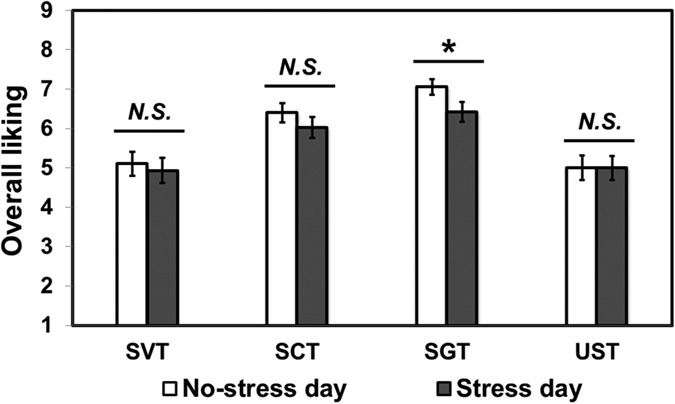
Comparisons of overall liking between “No-stress day” and “Stress day” for stevia-sweetened tea (SVT), sucralose-sweetened tea (SCT), sugar-sweetened tea (SGT), and unsweetened tea (UST). A Wilcoxon Signed-Rank test was used for data analysis. *N.S.* represents no significant difference at *P* < 0.05. *Represents a significant difference at *P* < 0.05. Error bars represent standard error of mean.

As shown in Fig. 5, only SGT showed significant differences in terms of overall liking between no-stress day and stress day. More specifically, SGT was less liked on stress day compared to no-stress day (*P* = 0.04). No significant differences were found for any other tea sample (*P* > 0.05, for all).

### Tea-induced calmness and pleasantness relationships for each tea sample with respect to sweetness intensity and overall liking

Previous research has shown strong correlations between food and beverage liking with calm and pleasant emotions^15^. For example, in a study conducted by Ng *et al*.^15^, consumers were asked to evaluate both their liking and emotional responses to 11 commercial blackcurrant squashes. When emotional responses were measured using a psychological literature-based emotion lexicon, i.e., EsSense Profile, consumers’ liking ratings showed positive correlations with calm (*r* = 0.782) and pleasant (*r* = 0.931) emotions, suggesting that people feel more calm and pleasant when they consume beverages that they like more. Thus, it was determined whether participants’ tea-induced calmness or pleasantness is related to their liking of tea samples. In addition, it was tested whether participants’ tea-induced calmness or pleasantness is associated with their sweetness intensity of tea samples.

As shown in Table 1, tea-induced calmness and pleasantness were not significantly associated with sweetness intensity of three tea samples (*P* > 0.05, for all), with the exception of SVT. SVT-induced calmness showed significant negative correlation with sweetness intensity on stress day (*rho* = −0.29, *P* = 0.045), but not on no-stress day (*rho* = −0.15, *P* = 0.28). Additionally, SVT-induced pleasantness showed significantly negative correlation with sweetness intensity on no-stress day (*rho* = −0.32, *P* = 0.02), but not on stress day (*rho* = −0.26, *P* = 0.064).

Interestingly, significant correlation was found between tea-induced calmness and overall liking of SVT (*rho* = 0.55, *P* < 0.001), SCT (*rho* = 0.48, *P* < 0.001), and UST (*rho* = 0.58, *P* < 0.001) on no-stress day, as well as on stress day: SVT (*rho* = 0.71, *P* < 0.001), SCT (*rho* = 0.39, *P* = 0.005), and UST (*rho* = 0.65, *P* < 0.001), respectively. However, SGT-induced calmness showed significant correlation with overall liking on stress day (*rho* = 0.31, *P* = 0.03) but not on no-stress day (*rho* = 0.19, *P* = 0.20). With respect to correlations between tea-induced pleasantness and overall liking, significant results were found for SVT (*rho* = 0.41, *P* = 0.003) and UST (*rho* = 0.46, *P* < 0.001) on no-stress day, as well as on stress day (*rho* = 0.49, *P* < 0.001, *rho* = 0.48, *P* < 0.001, respectively). SCT-induced pleasantness showed significant correlation with overall liking on no-stress day (*rho* = 0.49, *P* < 0.001), but not on stress day (*rho* = 0.22, *P* = 0.13). In contrast, SGT-induced pleasantness showed no correlations with overall liking of tea sample (*P* > 0.05, for both days).

## Discussion

This study was designed to determine a possible association between nutritive and non-nutritive sweeteners with emotional state of participants exposed to acute stressor in terms of tea-induced calmness and pleasantness. It should be noted here that calmness and pleasantness provide information on both attributes of emotional state, i.e., arousal and valence, respectively^16^. In addition, the association between acute stress and sweet taste perception of both types of sweeteners was determined with respect to intensity and liking. In the present study, acute stress was induced in participants by requiring them to solve a timed IQ test based on math and logic problems. Results comparing POMS ratings on both stress and no-stress days confirm that the method used was effective in imparting acute stress. This finding was consistent with that of previous studies that have also effectively used an IQ test as a stress inducer^17^.

The results from this study show that association of sweeteners with the emotional state of participants exposed to acute stressor varies by the caloric content of sweetener (i.e., nutritive versus non-nutritive). More specifically, as shown in Fig. 2, tea-induced calmness (i.e., calmness rating after drinking tea sample – calmness rating before drinking tea sample) differed with the sweetener used. Interestingly, drinking tea sweetened with sugar (nutritive sweetener) increased calmness under acute stress, but this trend was neither obtained when drinking tea sweetened with stevia or sucralose, nor when drinking unsweetened tea.

This study indicates that sugar-induced calmness under acute stress is not related to the sweetness intensity of sweetened tea. As shown in Fig. 4, acute stress has no substantial impact on sweetness intensity of nutritive or non-nutritive sweeteners, which is similar to the results reported in other studies^2^. It is therefore concluded that the difference between nutritive and non-nutritive sweeteners with respect to tea-induced calmness may not result from sweetness intensity between both types of sweeteners. This idea is supported by the lack of a significant correlation of tea-induced calmness with sweetness intensity in almost all cases (Table 1).

Our findings demonstrate that the difference between nutritive and non-nutritive sweeteners with respect to tea-induced calmness is not fully explained by overall liking of tea samples. More specifically, as shown in Fig. 5, it is worth noting that overall liking of sugar-sweetened tea was significantly lower under stress condition than under no-stress condition. However, overall liking of tea samples did not differ between under no-stress and stress conditions in either unsweetened tea or sweetened tea samples with non-nutritive sweeteners. In addition, as shown in Table 1, the association between tea liking and tea induced calmness was found to be more pronounced in either unsweetened tea or sweetened tea samples with non-nutritive sweeteners (stevia or sucralose) when compared to in sugar sweetened tea. In other words, sugar-induced calmness under acute stress might not be directly related to overall liking of sugar-sweetened tea. An explanation of these findings can be found in the concept of “food reward”. The food reward comprises of two functional components such as “wanting” (motivation to eat) and “liking” (hedonic impression)^18^. Both these components have been reported to follow separate pathways to affect the food reward^18^. While “wanting” component seems to be associated with mesotelencephalic dopamine neurotransmitter systems and the central nucleus of the amygdala, the “liking” component appears to be mediated by opioid and benzodiazepine/gamma-aminobutyric acid (GABA) neurotransmitter systems, and substantia innominata/ventral pallidal circuits^18^. Earlier research suggests that nutritive sweeteners have shown potential to cause central opoid release directly through taste pathways affecting the reward system^19^,^20^. However, similar effect of non-nutritive sweeteners has not been extensively reported probably because non-nutritive sweeteners activate taste pathway differently from nutritive sweeteners^21^. Therefore, nutritive sweeteners such as sugar might have a stronger relationship with the “liking” component of food reward compared to non-nutritive sweeteners. As a result, the lowered food reward perception due to acute stress^22^ may be manifested as reduced liking of nutritive sweetener, but not non-nutritive sweeteners among participants exposed to acute stressor. However, further scientific evidence is necessary for conclusive evidence in this regard.

How do we account for the difference between nutritive and non-nutritive sweeteners with respect to tea-induced calmness under acute stress? Brain imaging studies might explain such a difference based on the concept of “food reward”, especially the “wanting” component. Previous research showed that although both nutritive (sucrose) and non-nutritive (sucralose) sweeteners activated brain regions, such as the frontal operculum/insular, related to primary taste pathway, sucrose, but not sucralose, activated the brain regions of taste reward circuits, such as the left ventral striatum, left dorsal caudate nucleus, right thalamus, and bilateral midbrain at the significance level given in the study^21^. In other words, non-nutritive sweeteners may not be enough to fulfill food reward that results from caloric sweet ingestion^21^. This assumption has been supported by other brain imaging studies, which found that caloric drink consumption elicited more neural activation in the striatum, which is involved with food reward, than non-caloric drink consumption^23^,^24^. In addition, the difference between nutritive and non-nutritive sweeteners with respect to tea-induced calmness under acute stress can be explained from a physiological standpoint with respect to emotional state indicators such as cortisol level. When the effect of the two sweeteners, sugar and aspartame, on free cortisol concentration was compared in women, sugar consumption strongly reduced cortisol response under stress conditions compared to aspartame consumption^25^. Since lower cortisol response is associated with a calmer emotional state, it is possible that consuming food or beverages including nutritive sweetener (sucrose) results in a calmer emotional state. In other words, tea-induced calmness might not be due to sweet taste, but rather to caloric content. Stress-induced glucocorticoids such as cortisol encourage catabolism to provide fuel for the brain and the energy required during stress is restored, during recovery from stress, by promoting calorie intake, lipogenesis, and glycogen synthesis^25^,^26^. In this way, nutritive sweeteners, but not non-nutritive sweeteners, might reduce glucocorticoid-driven energy requirements for catabolism and mobilization of the energy stores by providing fuel to cater for the energetic demands of stress^25^. Previous rodent studies suggest that sucrose consumption switches off neural stress networks that mediate stress-induced hypothalamic-pituitary-adrenal (HPA), the autonomic nervous system, and emotional reactivity^25^,^27^. In addition, Tryon *et al*.^25^ found that consumption of beverages sweetened with sugar, but not aspartame, activated the hippocampus which according to Furay *et al*.^28^ is typically deactivated during acute stress.

It is also worth noting that there were no differences in tea-induced pleasantness (i.e., pleasantness rating after drinking tea sample – pleasantness rating before drinking tea sample) between under no-stress and stress conditions for all sweetened and unsweetened tea samples. These results are in line with previous studies that have shown no significant difference between consumptions of nutritive and non-nutritive sweeteners with respect to neural activation in the brain areas, such as amygdala, associated with pleasantness evaluation^23^. Smeets *et al*.^23^ suggested the lack of difference between consumptions of nutritive and non-nutritive sweeteners to be due to amygdala habituation responses to repeated orosensory stimulation with sweet taste^29^.

To summarize, this study shows that the association between sweeteners and emotional state of participants exposed to acute stressor may be more pronounced for nutritive sweeteners such as sugar than for non-nutritive sweeteners such as stevia and sucralose. This association is stronger for the arousal attribute of emotional state than with the valence attribute. More specifically, a consumption of tea sweetened with nutritive sweetener, but not non-nutritive sweetener, increased calmness of participants exposed to acute stressor while this finding is not fully explained in terms of sweetness intensity and overall liking of each sweetened tea samples. Calmness induced by consuming sugar-sweetened tea under acute stress might not result from sweet taste, but from calorie of the sweetener. Currently, from the point of view lifestyle prophylaxis, people tend to avoid sugar with calorie. This study demonstrated, however, sugar-sweetened tea had sedative effect for consumers with acute stress. These effects might be specific character of nutritive sweetener with calorie, not of non-nutritive sweetener.

## Methods

This study was conducted in accordance with the Declaration of Helsinki for studies on human participants. The protocol used in this study was approved by the Institutional Review Board of the University of Arkansas (Fayetteville, AR, USA). Prior to participation, the experimental procedure was explained to all participants and a written informed consent was obtained from each.

### Participants

Fifty healthy adults (25 men and 25 women) ranging in age from 22 to 70 years (mean age ± standard deviation = 41 ± 14 years) were recruited through a consumer profile database from the University of Arkansas Sensory Service Center (Fayetteville, AR, USA) that inherently contains information about more than 6,200 Northwest Arkansas residents. Participants were screened by their preference for and frequency of drinking both unsweetened and sweetened tea. Participants who did not like or never drank either of these beverages were not recruited for the study. Additionally, participants who never used artificial sweeteners in their beverages were not selected for the study. Since this was a study regarding acute stress, participants suffering from chronic stress were not included, i.e., participants with Perceived Stress Scale (PSS)^30^ scores > 25 were excluded from the study.

### Sample Preparation

Unsweetened instant tea powder (Lipton, Unilever, Englewood Cliffs, NJ) was used to prepare the tea beverages. Unsweetened tea sample (UNT) was prepared according to package instructions, without the addition of any sweetener. Three types of sweeteners were used in this study: pure cane sugar (Great Value, Wal-Mart Stores, Inc., Bentonville, AR), granulated no calorie sucralose (Great Value, Wal-Mart Stores, Inc., Bentonville, AR), and granulated no calorie stevia (Great Value, Wal-Mart Stores, Inc., Bentonville, AR). Sugar-sweetened tea sample (SGT) was prepared at 5% (w/v) concentration in spring water (Mountain Valley Springs Co., LLC Hot Springs, AR) according to package instructions. Sucralose-sweetened tea sample (SCT) and stevia-sweetened tea sample (SVT) were similarly prepared at 5% sucrose equivalent concentrations, which means perceived intensity of each sample was same as 5% (w/v) sucrose solution as indicated by manufacturers’ instructions. This level of sweetness concentration has been used in prior studies^3^ and was found to be acceptable based on pilot studies done by the authors of this study. In addition, unsweetened tea sample (UST) was prepared according to package instructions, without addition of any sweetener. The primary purpose of including UST was as a control sample. Many studies use water as a control^3^,^21^, but in this study using water would make it difficult to separate the sole impact of sweeteners from that of tea.

### Procedure

The study was conducted over a span of two days, one week apart. Each participant attended a “no-stress day” session and a “stress day” session. Day 1 was no-stress day for half of the participants, while the other half experienced it as stress day, and vice versa with respect to Day 2. A brief orientation was given each day to introduce the purpose of the study and explained the experimental procedure. Researchers also highlighted that participants should focus on evaluating the sweetness of samples and try to eliminate the influence of any taste preference bias.

#### No-stress day

On no-stress day, participants were asked to complete a reduced version of a Profile of Mood States (POMS)^14^ questionnaire after the orientation session. Participants rated their each mood on a 5-point scale ranging from 1 (very slightly) to 5 (extremely). Participants were then seated in individual sensory booths to taste the tea samples. A total of four samples were presented in a sequential monadic fashion based on William Latin Square Design^31^. Approximately 90-mL of each sample was provided in a 112-mL cup with a three-digit code. Participants were asked to rate on a 9-point scale how calm (1: extremely stressed; 9: extremely calm) and pleasant (1: extremely unpleasant; 9: extremely pleasant) they felt before drinking the tea samples. Participants were then asked to drink the entire cup of tea and rate only its sweetness intensity on a 15-cm anchored line scale (0: extremely weak; 15: extremely strong). Participants also rated their overall liking of the tea sample on a 9-point hedonic scale (1: dislike extremely; 9: like extremely). Participants also rated how calm and pleasant they felt after drinking the tea sample similar to how they did before drinking the sample.

#### Stress day

On stress day, participants were asked to complete a reduced version of a POMS questionnaire after the orientation session. Participants were then seated in individual sensory booths and asked to complete an intelligence quotient (IQ) test (19 questions) within a time limit of 10 minutes. Participants were told that the scores of the IQ test were to be used to divide them into “Low”, “Medium”, or “High” IQ groups. The questions included in the IQ test were moderate-to-difficult math and logic problems. Participants were required to choose the correct answer from four given choices. The purpose of the IQ test was to induce acute stress into participants, and it had been found to be successful in this regard during pilot testing. Similar methods have been used in previous studies as well^3^,^17^,^32^. After the stressor, i.e., the IQ test, participants filled out the POMS questionnaire once again. Participants then performed evaluation following the same procedure as on no-stress day.

### Data Analysis

Data was collected using Compusense five (Release 5.6, Compusense Inc., Guelph, ON, Canada) software and analyzed using SPSS 22.0 for Windows (IBM SPSS Inc., Chicago, IL). A Shapiro-Wilk test revealed that sweetness intensity, overall liking, tea-induced calmness (i.e., calmness rating after drinking tea sample – calmness rating before drinking tea sample), tea-induced pleasantness (i.e., pleasantness rating after drinking tea sample – pleasantness rating before drinking tea sample), and POMS ratings were not normally distributed (*P* <  0.05, for all). Therefore, a Wilcoxon Signed-Rank test was used to compare the sweetness intensity, overall liking, tea-induced calmness, and tea-induced pleasantness among the four tea samples on stress day and no-stress day. To verify whether the stressor (IQ test) was effective in inducing acute stress, a mean anxiety score was calculated by averaging the POMS ratings for the following emotions^33^: “anxious”, “nervous”, “on edge”, “panicky”, “restless”, “relaxed”, “shaky”, “tense”, and “uneasy”. Mean anxiety scores (score range: 1 to 5) for three combinations were compared using a Wilcoxon Singed-Rank test: “stress day after the IQ test” versus “no-stress day”, “stress day before the IQ test” versus “no-stress day”, and “stress day before the IQ test” versus “stress day after the IQ test”. In addition, three individual positive moods (calm, cheerful, relaxed; score range: 1 to 5, respectively) and four negative moods (annoyed, anxious, stressed, and tensed; score range: 1 to 5, respectively) were compared between stress day after the IQ test and no-stress day using a Wilcoxon Signed-Rank test. A statistically significant difference was defined when *P* < 0.05. Spearman’s correlation analyses were used to examine relationships of tea-induced calmness and pleasantness of each tea sample with respect to its sweetness intensity and overall liking; a statistically significant correlation was defined when *P* < 0.05.

## Additional Information

**How to cite this article**: Samant, S. S. *et al*. Tea-induced calmness: Sugar-sweetened tea calms consumers exposed to acute stressor. *Sci. Rep.* **6**, 36537; doi: 10.1038/srep36537 (2016).

**Publisher’s note:** Springer Nature remains neutral with regard to jurisdictional claims in published maps and institutional affiliations.

## Figures and Tables

**Table 1:** Spearman’s correlation coefficients in the relationships of sweetness intensity and overall liking with tea-induced calmness and pleasantness in stevia-sweetened tea (SVT), sucralose-sweetened tea (SCT), sugar-sweetened tea (SGT), and unsweetened tea (UST) on “no stress day” and “stress day”.

	Tea-induced calmness		Tea-induced pleasantness	
	no-stress day	stress day	no-stress day	stress day
*Sweetness intensity*				
SVT	−0.15	−0.29*	−0.32*	−0.26
SCT	0.03	0.06	0.01	−0.18
SGT	−0.12	−0.09	−0.06	−0.07
UST	−0.02	0.04	−0.19	−0.06
*Overall liking*				
SVT	0.55***	0.71***	0.41**	0.49***
SCT	0.48***	0.39**	0.49***	0.22
SGT	0.19	0.31*	0.10	0.16
UST	0.58***	0.65***	0.46***	0.48***

*,** and ***represent a significance at *P* < 0.05, *P* < 0.01 and *P* < 0.001, respectively.
